# A High Content Screening Assay to Identify Compounds with Anti-Epithelial-Mesenchymal Transition Effects from the Chinese Herbal Medicine Tong-Mai-Yang-Xin-Wan

**DOI:** 10.3390/molecules21101340

**Published:** 2016-10-10

**Authors:** Ningning Liu, Lailai Li, Xin Zhu, Zhiqiang Ling, Jianguo Feng, Ying Hu, Yi Wang, Lijun Mou, Yi Wang

**Affiliations:** 1 TCM Research Center, Tianjin University of Traditional Chinese Medicine, Tianjin 300193, China; 18867141533@163.com (N.L.); mooching@126.com (L.L.); vip_wangyi@126.com (Y.W.); 2 Zhejiang Cancer Research Institute, Zhejiang Cancer Hospital, Hangzhou 310022, China; zhuxin@zjcc.org.cn (X.Z.); lingzq@hotmail.com (Z.L.); fengjg@zjcc.org.cn (J.F.); 3 Department of Nephrology, The Second Affiliated Hospital, College of Medicine, Zhejiang University, Hangzhou 310029, China; huyinghz@126.com; 4 College of Pharmaceutical Sciences, Zhejiang University, Hangzhou 310058, China

**Keywords:** high-content screening, epithelial mesenchymal transition, fluorescence imaging

## Abstract

Chronic kidney disease (CKD) is a worldwide health problem with growing prevalence in developing countries. Renal tubular epithelial-mesenchymal transition (EMT) is a critical step and key factor in the development of this condition. Renal tubulointerstitial fibrosis is a basic pathological change at the later stages of the disease. Therefore, blocking the development of EMT could be a critical factor in curing CKD. We have established a cell-based high-content screening (HCS) method to identify inhibitors of EMT in human proximal tubular epithelial (HK-2) cells by automatic acquisition and processing of dual-fluorescent labeled images. With the aid of chromatographic separation and mass spectrometry, we achieved the rapid and reliable screening of active compounds from the Chinese herbal medicine Tong-Mai-Yang-Xin-Wan (TMYX) for treating EMT. Five fractions were found to exert anti-EMT activity and were further identified by liquid chromatography coupled with tandem mass spectrometry. Glycyrrhizic acid, glyasperin A, and licorisoflavan A were found to inhibit EMT. The proposed approach was successfully applied to screen active compounds from TMYX on TGF-β1-stimulated HK-2 cells and may offer a new means for identifying lead compounds for treating EMT from registered Chinese herbal medicines.

## 1. Introduction

Chronic kidney disease (CKD) is a worldwide health problem, and its incidence and mortality have increased in recent years. Inflammation is the response of vascularized living tissue to a variety of stimuli. Chronic inflammation is acknowledged to be pivotal in the development and progression of CKD. When kidneys are exposed to prolonged inflammatory stimuli, the evaluated release of transforming growth factor (TGF-β) eventually activates the tubular epithelial-mesenchymal transition (EMT) [1]. Many mediators of inflammation have been discovered in adults with CKD and end-stage nephropathy [2]. Renal tubulointerstitial fibrosis is a basic pathological change in end stage nephropathy [3], whereas EMT is the critical step and key factor during this process. The loss of adhesive structure of epithelial cells and gain of migratory capacity of mesenchymal cells are pivotal characteristics of EMT. Additionally, the excessive deposition of extracellular matrix, which disrupts the normal structure, is an important aspect of the process [4]. The current therapeutic methods for treating CKD include dialysis [5], transplantation [6], as well as some indirect strategies, such as alkali therapy [7] and statin therapy [8,9]. However, clinical therapeutics cannot block or reverse the EMT process in early stage CKD. Therefore, identifying lead compounds with anti-EMT effects from Chinese herbal medicines is of extremely high relevance.

Chinese herbal medicines are regarded as invaluable resources in lead compound discovery. In recent years, many herbs or herbal remedies have been found to produce resistance against EMT. The compounds baicalin and baicalein extracted from *Scutellaria baicalensis* Georgi reduced the expression levels of EMT-related transcription factors and inhibited TGF-β1-induced EMT in MCF10A cells [10]. Resveratrol, a well-known botanical drug derived from red grapes, was reported to exert anti-EMT efforts by inhibiting the activation of hedgehog signaling in vitro and in vivo [11]. Amygdalin can suppress keloid fibroblast cell proliferation and also inhibit TGF-β1 secretion in lymphocytes [12]. Several active compounds isolated from Chinese herbal medicines, such as salvianolic acid B [13,14,15], curcumin [16], and norcantharidin [17] were reported to prevent TGF-β1-induced proliferation in human proximal tubule epithelial (HK-2) cells. However, the discovery of these compounds has been chiefly based on time-consuming pharmacological studies, which is not a suitable approach for large-scale screening. Therefore, developing an appropriate assay to rapidly screen anti-EMT compounds is necessary.

With the aid of fluorescent microscopy and automated image capturing and processing technology, high-content screening (HCS) has become an emerging technique in drug discovery. HCS is an integrated cell biological investigation approach with image acquisition, processing, and analysis. This method differs from the traditional manual or semi-automated image acquisition and data extraction [18]. In recent years, a high-content whole-well imaging approach has been established to screen compounds against drug-induced cardiotoxicity [19]; and drug-induced human hepatotoxicity [20]. This kind of screening also includes the identification of novel nuclear export inhibitors [21], as well as compounds that detect hepatotoxicity in HepG_2_ cells [22]. To the best of our knowledge, only a few reports related to HCS for screening compounds with anti-EMT effects have been published. For example, Maier used a high-content imaging method to monitor and quantify the dynamic changes of endogenous vimentin by developing vimentin-specific nanobodies [23]. The study provided basis for screening novel therapeutics that affect EMT.

Tong-Mai-Yang-Xin-Wan (TMYX) is a common Chinese medicine, approved by the State Food and Drug Administration of China with approval number Z12020589. TMYX has been widely used in treating cardiovascular diseases and other inflammation-related diseases. This Chinese medicine consists of 11 herbs, including *Rehmannia glutinosa* Libosch, *Spatholobus suberectus* Dumn, *Ophiopogon japonicus*, *Glycyrrhiza uralensis* Fisch, *Polygonum multiflorum* Thunb, *Equus asinus* L, *Schisandra chinensis*, *Codonopsis pilosula*, *Chinemys reevesii*, *Ziziphus jujuba* Mill and *Cinnamomum cassia* Presl. In the Chinese pharmacopeia of People’s Republic of China, glycyrrhizic acid is regarded as an indicator in the content determination of TMYX. In our preliminary study, we found several compounds with dose-dependent anti-inflammatory effects in TMYX [24]. Therefore, screening active compounds from TMYX is a vital endeavor for CKD drug discovery.

In the present study, a rapid and reliable assay was established to screen anti-EMT compounds from TMYX. HCS technology coupled with chromatographic separation and mass spectrometry can rapidly screen active ingredients from complex mixtures. The expression of the α-smooth muscle actin (α-SMA), a commonly used EMT biomarker, was monitored in TGF-β1-stimulated HK-2 cells. The appropriable dosage and stimulation time of TGF-β1 were optimized. The proposed approach was successfully applied in screening active components from TMYX. Five fractions were found to exert anti-EMT activity and were further identified by liquid chromatography (LC) coupled with mass spectrometry (MS). The anti-EMT effects of glycyrrhizic acid, glyasperin A, and licorisoflavan A were further validated by in vitro assays.

## 2. Results and Discussion

### 2.1. Development of the HCS Assay for Screening Compounds with Anti-EMT Effects

**Figure 1:**
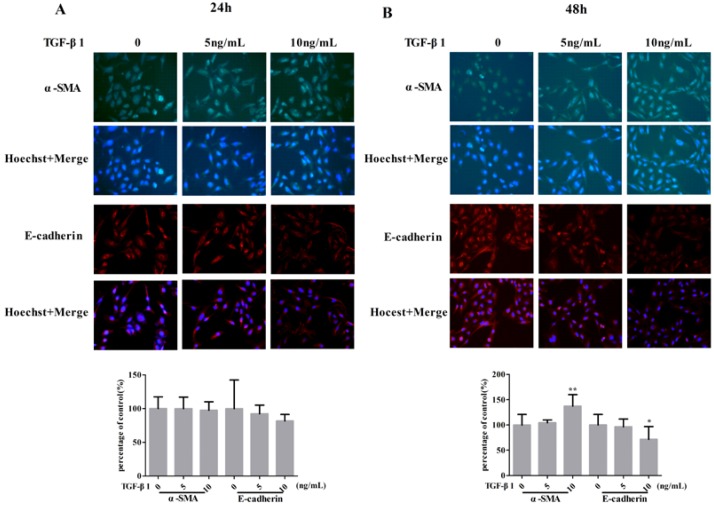
Expression of α-SMA and E-cadherin in HK-2 cells after stimulated by TGF-β1 in different concentrations for 24 h (**A**) and 48 h (**B**). The error bars represent standard deviation based on three repeated. All data was represented by Mean ± SD, ** *p* < 0.01, * *p* < 0.05, *n* = 12 (single-trial).

HCS is a technique that screens compounds by a cell-based and microscopy-image-based assay in a microwell plate. After the compounds stimulate the cells for an appropriate time, high-content images can be obtained by detecting of fluorescent markers with automated microscopy. To establish a reliable HCS assay for screening active compounds with anti-EMT effects, visualization of the EMT process was optimized in HK-2 cells. First, the suitable dose of TGF-β1 and the appropriate time needed to induce significant characteristics of EMT were evaluated. HK-2 cells were stimulated with different concentrations of TGF-β1 (5 and 10 ng/mL) for different durations (24 and 48 h). The expression alteration of α-SMA and epithelial adhesion molecule (E-cadherin), two commonly used EMT biomarkers, were monitored by fluorescent microscopy. After stimulation of TGF-β1 for 24 h, no significant change in fluorescence intensity was observed for α-SMA and E-cadherin (Figure 1A). However, the fluorescent images in Figure 1B show that TGF-β1 at 10 ng/mL concentration exhibits prominent attenuation of the fluorescence intensity of E-cadherin and enhances α-SMA expression after incubation for 48 h. To control the effect of TGF-β1 or compounds on the total numbers of cells, all data on fluorescence intensity were normalized to Hoechst staining, which was used to stain the nucleus. Many documents have stated that either of the two biomarkers can be used to confirm EMT. Therefore, in our entire experiment, the α-SMA biomarker was used to screen EMT inhibitors.

**Figure 2:**
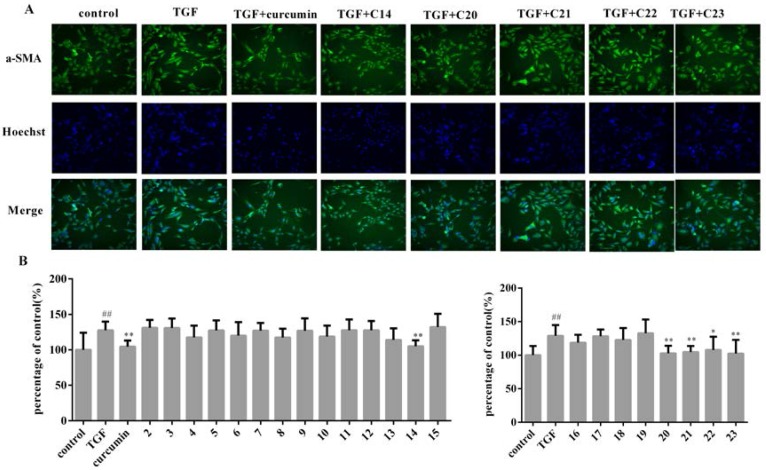
The screening results of anti-EMT fractions from TMYX. The fluorescence images of active components **14**, **20**, **21**, **22**, **23** (**A**) and the fluorescence intensity of components **2**–**23** from TMYX (**B**). The error bars represent standard deviation based on three repeated. All data was represented by Mean ± SD, ^##^ *p* < 0.01, ** *p* < 0.01, * *p* < 0.05, *n* = 12 (single-trial).

The proposed approach was successfully applied in screening anti-EMT activity from 22 fractions of TMYX. After exposed to TGF-β1, the morphology of the cells shrank whilst the treatment of the positive drug curcumin can restore the cell shape to normal condition. Representative images of HK-2 cells incubated with different active components are displayed in Figure 2A. The relative fluorescent intensities of 22 fractions from TMYX are listed in Figure 2B. When stimulated by TGF-β1, α-SMA expression increased by about 30% compared with the control group. After curcumin was added, α-SMA expression decreased by about 20% with respect to that of the TGF-β1 group. By comparing with the efficacy of curcumin, five fractions, including **C14**, **C20**, **C21**, **C22**, and **C23** were found with significant activity against TGF-β1-induced EMT. These fractions successfully reduced the expression levels of α-SMA by 17%, 20%, 16%, 18% and 20%, respectively.

Biology- and target-based approaches are commonly used as drug discovery strategies [25,26]. However, the biology-based approach is labor-intensive, time-consuming, and is not suitable for large-scale screening. Moreover, target-based approaches also have some limitations. Unlike these methods, the proposed HCS assay can rapidly identify active components from dozens or hundreds of samples through the automatic acquisition and processing of dual-fluorescent labeled images. The assay hence provides a novel means for determining lead compounds from Chinese herbal medicines.

### 2.2. Chemical Composition and Anti-EMT Activity of Active Components

**Figure 3:**
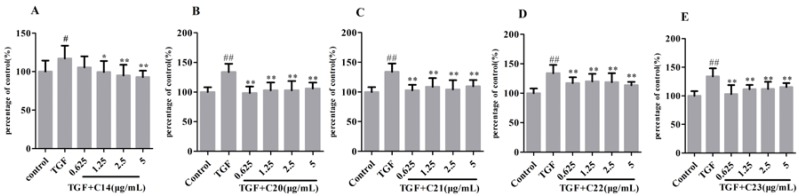
The dose-dependent anti-EMT activities of components **14** (**A**); **20** (**B**); **21** (**C**); **22** (**D**); **23** (**E**) from TMYX. The error bar represent standard deviation based on three repeated. All data was represented by Mean ± SD. Compared to the control, ^##^ *p* < 0.01, ^#^ *p* < 0.05. Compared to TGF-stimulated cells, ** *p* < 0.01, * *p* < 0.05, *n* = 12 (single-trial).

Five active fractions from TMYX were further validated in a dose-dependent manner, and the chemical compositions of these fractions were determined by LC-MS analysis. In Figure 3, fraction **14** shows an obvious inhibitory effect against EMT in the dose range of 1.25 μg/mL to 5 μg/mL.

**Figure 4:**
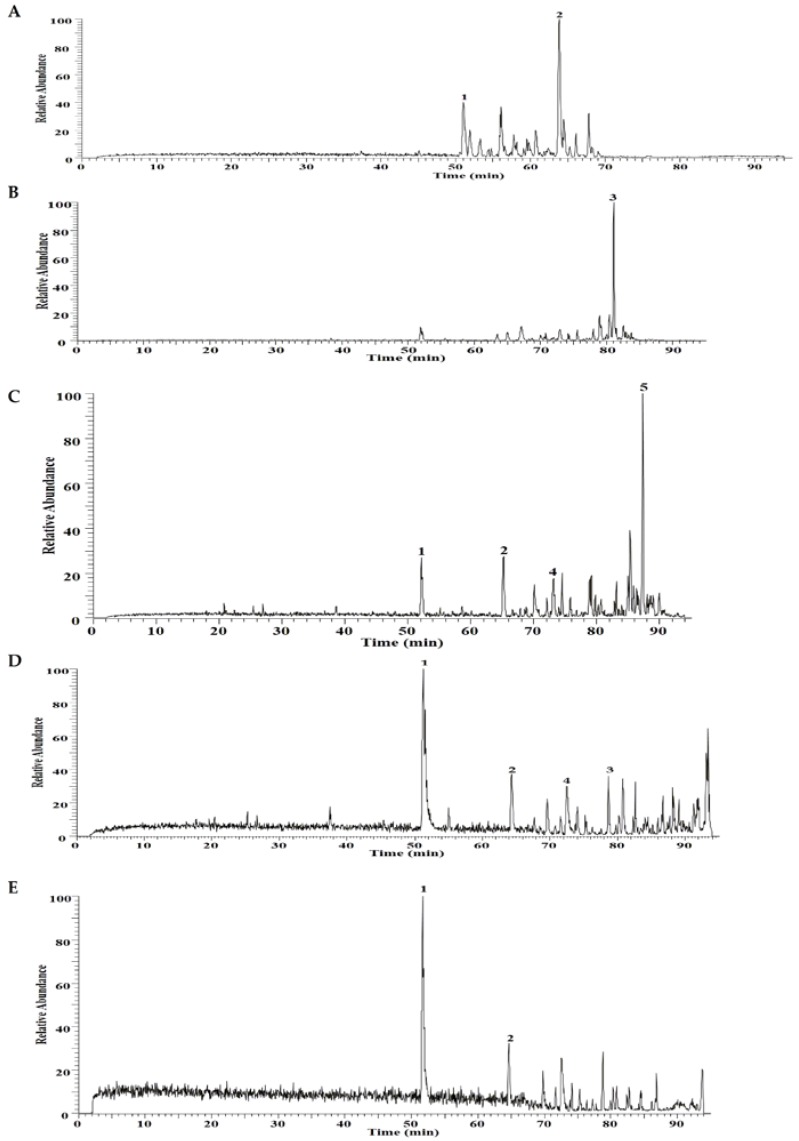
The chromatograms of fractions **14** (**A**); **20** (**B**); **21** (**C**); **22** (**D**); **23** (**E**) from TMYX. Five compounds including glycyrrhizic acid (**1**), glycycoumarin (**2**), glyasperin A (**3**), emodin (**4**), licorisoflavan A (**5**) were identified, respectively.

Meanwhile, the fractions **20**, **21**, **22**, and **23** display stable inhibitory effects on TGF-β1-induced EMT from 0.625 μg/mL to 5 μg/mL. The chromatograms of these active fractions are shown in Figure 4. Considering that these fractions have similar chemical compositions, we speculate that the shared compounds in these fractions may contribute to the anti-EMT effect in TMYX.

The LC-MS identifications of these compounds are summarized in Table 1. Five active fractions are found in the primary screening. Several compounds are found in each active fraction. We then selected five compounds that exhibited relatively high contents in these active fractions for further investigation, including emodin, glycyrrhizic acid, glycycoumarin, glyasperin A, and licorisoflavan A. Glycyrrhizic acid, glycycoumarin, glyasperin A and licorisoflavan A were isolated from *G. uralensis* Fisch. Some researchers have reported that extracts from *G. uralensis* Fisch exert a potential antidepressant-like effect on chronic-variable-stress-induced depression model rats [27], and may serve as antiviral components against EV71 and CVA16 infection [28]. However, the anti-EMT activity from the extracts of *G. uralensis* Fisch has rarely been reported. Emodin is an anthraquinone compound, which has been documented to play a role in treating kidney fibrosis [29,30]. However, the action mechanism of emodin for such treatment is unknown. Our results suggest that the anti-EMT effects of emodin may contribute to its effects on diabetic nephropathy by inhibiting the mesenchymal accumulation.

**Figure 5:**
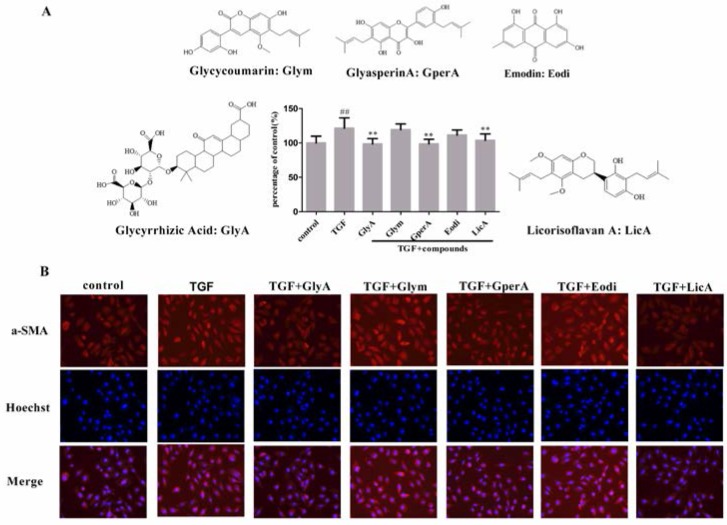
The structures of identified active compounds from TMYX against TGF-β1 induced EMT (**A**) and representative images of α-SMA expression (**B**). The error bars represent standard deviation based on three independent experiments. All data was represented by mean ± SD, ^##^ *p* < 0.01, ** *p* < 0.01, *n* = 12 (single-trial).

The effect of the identified compounds on α-SMA expression was further investigated by the proposed HCS assay. In this section, AlexaFluor 555 goat anti-mouse IgG (H + L) (1:200) was used to mark α-SMA. After stimulation by TGF-β1 in the absence of compounds, α-SMA expression increased by about 20% compared with the control group (Figure 5A). 

Through changes in fluorescence intensity, we noted that glycyrrhizic acid, glyasperin A, and licorisoflavan A decreased the α-SMA expression by about 20% in 1 μΜ. By contrast, no obvious influence in α-SMA expression was found between glycycoumarin and emodin. The corresponding fluorescence images are shown in Figure 5B. Chronic inflammation is closely associated with nuclear factor κB (NF-κB) activation, which may cause CKD development by increasing TGF-β levels [1]. Therefore, the anti-EMT activity of glycyrrhizic acid, glyasperin A, and licorisoflavan A in treating CKD was potentially achieved by the anti-inflammatory effects.

### 2.3. Validation of Anti-EMT Activities of Active Compounds by Confocal Microscopy

**Figure 6:**
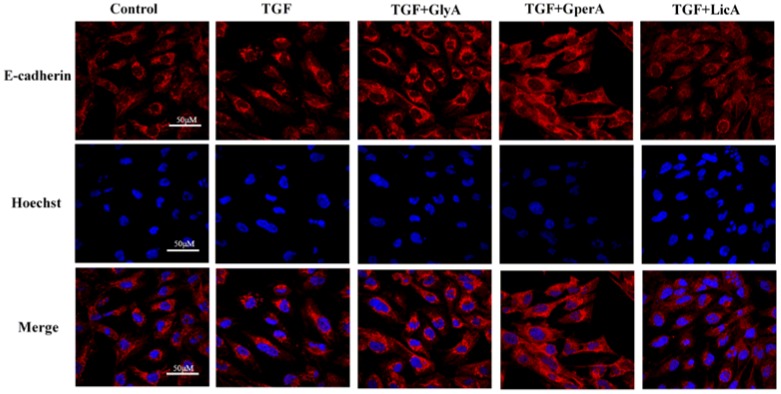
The effect of glycyrrhizic acid (GlyA), glyasperin A (GperA) and licorisoflavan A (LicA) on E-cadherin expression by confocal miscroscopy. Scale bar: 50 μM. *n* = 12 (single-trial).

Validation assays were applied to verify the anti-EMT effects of glycyrrhizic acid, glyasperin A and licorisoflavan A in TMYX recognized in the pre-stage test. E-cadherin is the well-studied member of the cadherin family. E-cadherin downregulation decreases the strength of cellular adhesion within a tissue, and results in an increase in cellular motility that promotes EMT progression. As the prototypical epithelial cell marker of EMT [31], E-cadherin was used to evaluate the screened compounds. In Figure 6, glycyrrhizic acid, glyasperin A, and licorisoflavan A prominently improve E-cadherin expression at 1 μM. This finding is consistent with previous results. Glycyrrhizic acid, glyasperin A, and licorisoflavan A display the ability to resist the EMT process, which might be used to treat CKD in the future.

## 3. Materials and Methods

### 3.1. Reagents and Chemicals

TGF-β1 was purchased from UcallM Biotechnology Co. (Wuxi, China). Rat tail tendon collagen type I was purchased from Solarbio (Beijing, China). The antibody against the epithelial adhesion molecule E-cadherin was purchased from Huabio (Hangzhou, China, murine monoclonal antibody, catalog number M1405-3,). The antibody against α-SMA was purchased from Sigma-Aldrich Crop (St. Louis, MO, USA, murine monoclonal antibody, catalog number A2547). AlexaFluor 555 goat anti-mouse IgG (H + L) (catalog number A0460) and fluorescein isothiocyanate (FITC) goat anti-mouse IgG (H + L) were obtained from Beyotime (Shanghai, China, catalog number A0568). Hoechst 33342 obtained from Sigma-Aldrich Crop was dissolved in dimethyl sulfoxide (DMSO) (Sigma) and stored at −20 °C before use. The cell culture reagents employed included Dulbecco’s modified Eagle medium F-12 Nutrient Mixture (DMEM/F-12), fetal bovine serum (FBS), and antibiotics, which were all purchased from Gibco (Grand Island, NY, USA). Curcumin, emodin, and glycyrrhizic acid standards were purchased from Shanghai Winherb Medical Technology Co. Ltd. (Shanghai, China). The standards of licorisoflavan A, glycycoumarin, and glyasperin A were purchased from Shanghai Yuanye Bio-Technology Co. Ltd. (Shanghai, China).

### 3.2. Cell Cultures and HCS Assays

HK-2 cell lines derived from human lines were obtained from the Zhejiang University of Chinese Medicine and maintained in DMEM/F-12 with 10% FBS and antibiotics (100 units/mL penicillin and 100 μg/mL streptomycin). When the cell population reached 70%–80%, the cells were seeded at a density of 3000 cells per well in 96-well plates in 100 μL for 1 day. The density was ensured suitable to withstand the repeated wash cycles in the subsequent experiment. Then, the medium was changed to a fresh serum-free medium with or without TGF-β1 (10 ng/mL) in the presence of samples for another 2 days. Curcumin, an active compound known to exert anti-EMT effects [32,33,34,35], served as the positive control drug.

Before being fixed in cold 4% paraformaldehyde, the cell monolayers were washed with PBS to remove dead cells. After fixing for 30 min, the cells were permeabilized with 0.1% Triton X-100 for 10 min, blocked with 5% bovine serum albumin (BSA) for 1 h, and then incubated overnight with mouse anti-α-SMA antibody (1:400). After PBS washing, the cells were incubated with fluorescent dye, namely, FITC-labeled goat anti-rabbit IgG (H + L) (1:200) (Green) or AlexaFluor 555 goat anti-mouse IgG (H + L) (1:200) (Red) at room temperature for 30 min. These two kinds of fluorescent dyes were combined with antibody protein to form the fluorescent antibody. The α-SMA or E-cadherin expression was determined and quantified through fluorescence signal changes. Cell counts were assessed by Hoechst 33342 (1:1000) staining for 10 min, and the stained samples were subjected to high-content imaging analysis on MetaXpress Micro XL (Sunnyvale, CA, USA). The image acquisition and fluorescence intensity measurements were conducted by automatic scanning through the software of DFTT × C5, using a 20× objective. Each condition, which involved three replicates and eight images per well was captured, specifically, four images for FITC staining (or AlexaFluor 555 staining) and four images for Hoechst 33342. The protocol for E-cadherin was similar to that used for the AlexaFluor 555 goat anti-mouse IgG (H + L). The high-content imaging analysis can scan many culture plates in a short time and hence avoid the influence of fluorescence quenching, ensuring the credibility of the results. Owing to either of the two EMT biomarker can be used to confirm EMT, thus, in our entire screening experiment, the α-SMA biomarker was used to screen EMT inhibitors, and the E-cadherin biomarker was employed to verify the screened active compounds.

### 3.3. Preparation of Standard Compounds from TMYX

TMYX was crushed and then extracted twice with methanol. The extract solution was filtered, concentrated, and centrifuged at 13,400 r/min for 5 min. Then, the supernatant was purified through an octadecylsilyl column (ODS) medium-pressure column. First, pure water was used as eluent to remove the polysaccharide interferents, and then, the TMYX residue components were eluted with methanol. The methanol eluent was centrifuged, and the supernatant was obtained for standard preparation. The preparation conditions were as follows: Zorbax Stable Bond C18 (dimensions: 21.2 mm × 250 mm, 7 μm, Agilent, Santa Clara, CA, USA), with 0.1% formic acid solution (A) and acetonitrile (B) as mobile phase. A gradient program was adopted in line with the following profile: 0 min, 5% B; 60 min, 90% B; 72 min, 95% B; 82 min, 95% B. Finally, 22 fractions were obtained and then named **C2**–**C23** successively, and the other components were discarded. Afterward, the samples were heated in a water-bath for 24 h, freeze-dried for 24 h, and then dissolved in DMSO prior to cell treatment. All the wells were ensured to contain DMSO at less than 0.1%.

### 3.4. Screening of Anti-EMT Compounds from TMYX 

The tested samples of TMYX were added following the experimental protocol described above. In the first screening, the concentration of each of the 22 fractions extracted from TMYX was set to 25 μg/mL (except for some fractions that displayed cytotoxicity, namely fractions **14**–**23**; the concentrations of these fractions were set to the maximum non-toxic dosage. Full experimental details are given in the Supplementary Materials). Then, the active fractions with strong activity were selected, and their efficacies were validated from 0.625 μg/mL to 5 μg/mL in the second screening.

### 3.5. Identification of Active Components by LC-MS

The identification of the active component compounds of TMYX was reported to in our previous work [24]. The active components of TMYX were characterized by LC-MS. The chromatographic separation was conducted on an Agilent Zorbax SB-C18 column (4.6 mm × 250 mm, 5 μm). The mobile phases were 0.05% formic acid-water (A) and acetonitrile (B). The gradient program was as follows: 0–20 min, 10%–30% B; 20–40 min, 30%–35% B; 40–60 min, 35%–50% B; 60–80 min, 50%–80% B; 80–90 min, 80% B; 90–91 min, 80%–95% B; 91–95 min, 95% B. The acquisition parameters of the mass spectra were as follows: capillary temperature, 350 °C, sheath gas flow, 18 L/min; and auxiliary gas flow, 6 L/min. The samples were analyzed in positive and negative modes, respectively. The parameters of negative mode were as follows: ion source, 3 kV; source current, 80 μA; capillary voltage, −15 V; tube lens offset voltage, −30 V. The parameters of the positive mode were ion source, 4 kV; source current, 80 μA; capillary voltage, 19 V; and tube lens offset voltage, 25 V. Data were obtained at *m*/*z* 100–1500 in a full scan.

The accurate mass measurements were determined by TripleTOFTM5600+ (AB Sciex, Concord, ON, Canada). The chromatography conditions were the same as that of the LC-MS analysis described above. The parameters were as follows: nebulizer pressure, 60 psi; gas pressure, 25 psi; auxiliary gas pressure, 60 psi; ion spray voltage, −4.5 kV; and declustering potential, 100 V. The source temperature was 100 V, and the full scan acquisition mode was adopted.

### 3.6. Validation of Active Compounds with Anti-EMT Effects by Confocal Microscopy

HK-2 cells were cultured in collagen I-coated six-well plates. Then, glycyrrhizic acid, glyasperin A, and licorisoflavan A (1 μM) were added after 24 h in the presence of TGF-β1. The next procedures followed the experimental design we described above. The mouse anti-E-cadherin antibody (1:400) was incubated overnight, and then incubated with AlexaFluor 555 goat anti-mouse IgG (H + L) (1:200) for 30 min. The fluorescent images were obtained by an AIR laser scanning confocal microscope (Nikon, Tokyo, Japan) with a 60× lens. Each well captured 12 containing six images for AlexaFluor 555 staining and six images for Hoechst 33342. The fluorescence intensity was measured by ImageJ Software (version 6.0, National Institutes of Health, Bethesda, MD, USA).

### 3.7. Z’ Factor

In the high-throughput screening assay, the feasibility of some experimental conditions and measuring methods for the high-throughput experiment was first verified. The Z’ factor reflects both the assay signal dynamic range and data variation associated with signal measurements and is suitable for assay quality assessment [36]. The equation of the Z’ factor is:

Z’ factor = 1 − (3 × (σp + σn)/|(μp − μn)|)

where σ is the sample variance, μ is the average value, p is the positive control, and n is the negative control. In our experiment, curcumin was adopted as the positive drug, and the sample without treatment was used as negative control. Z’ factor between 0.5 and 1 reflects a high test result. In our test, the Z’ factor was 0.5 and showed that our method is suitable for high-throughput assay.

### 3.8. Statistical Analysis

All experiments were carried out for three independent replicates. GraphPad Prism (GraphPad Software Inc., La Jolla, CA, USA) was used for all statistical analysis with the statistical significance considered at *p* < 0.05. Differences between groups were tested by one-way ANOVA with Dunnett’s test. All data are expressed in terms of mean and standard deviation.

## 4. Conclusions

In the present study, a rapid and reliable HCS assay was established to screen active compounds with anti-EMT effects. With the aid of chromatographic separation and mass spectrometry identification, the proposed approach was applied to identify active components of a Chinese herbal medicine which could inhibit TGF-β1 induced EMT. Three active compounds, glycyrrhizic acid, glyasperin A and licorisoflavan A, were successfully identified. Given that inflammation is involved in the pathological process of CKD and other complications, compounds that can inhibit inflammation-associated cytokine production and reduce inflammatory reactions should be used to evaluate CKD treatment, therefore we conclude that the compounds glycyrrhizic acid, glyasperin A, and licorisoflavan A show anti-inflammation effects in CKD treatment. The presented approach may offer a rapid and reliable way to screen active compounds from Chinese herbal medicines for CKD treatment.

## Figures and Tables

**Table 1:** The identification of compounds from active components.

Peak No.	*t*_R_ (min)	Molecular Formula	[M − H]^−^/[M + H]^+^		Identification	Source
			Detected	Error (ppm)		
1	51.26	C_42_H_62_O_16_	821.3956	0.3	Glycyrrhizic acid	*Glycyrrhiza uralensis* Fisch
2	63.83	C_21_H_20_O_6_	367.1180	0.9	Glycycoumarin	*Glycyrrhiza uralensis* Fisch
3	81.07	C_25_H_26_O_6_	421.1639	−1.6	Glyasperin A	*Glycyrrhiza uralensis* Fisch
4	72.53	C_15_H_10_O_5_	269.0452	2.6	Emodin	*Rheum palmatum* L
5	87.27	C_27_H_34_O_5_	437.2321	−0.4	Licorisoflavan A	Glycyrrhiza uralensis Fisch

## Supplementary Materials

Supplementary materials can be accessed at: http://www.mdpi.com/1420-3049/21/10/1340/s1.
