# Supplementary figures and images for: A High Content Screening Assay to Identify Compounds with Anti-Epithelial-Mesenchymal Transition Effects from the Chinese Herbal Medicine Tong-Mai-Yang-Xin-Wan
Source: Molecules. 2016 Oct 10;21(10):1340. doi: 10.3390/molecules21101340 (PMC6273035; doi:10.3390/molecules21101340)

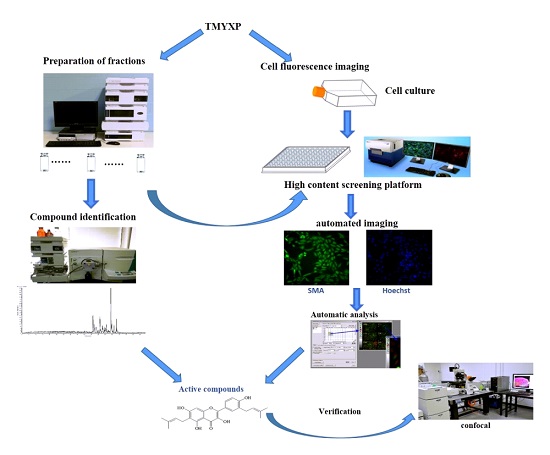

Supplement: Supplementary file 1 [file molecules-21-01340-s001.jpg]
